# In-Vitro Antibacterial Activity of Curcumin-Loaded Nanofibers Based on Hyaluronic Acid against Multidrug-Resistant ESKAPE Pathogens

**DOI:** 10.3390/pharmaceutics14061186

**Published:** 2022-05-31

**Authors:** Petr Snetkov, Elizaveta Rogacheva, Arina Kremleva, Svetlana Morozkina, Mayya Uspenskaya, Liudmila Kraeva

**Affiliations:** 1 Center of Chemical Engineering, ITMO University, Kronverkskiy Prospekt, 49, bldg. A, 197101 St. Petersburg, Russia; morozkina.svetlana@gmail.com (S.M.); mv_uspenskaya@itmo.ru (M.U.); 2 Saint-Petersburg Pasteur Institute, Street Mira, 14, 197101 St. Petersburg, Russia; elizvla@yandex.ru (E.R.); lykraeva@yandex.ru (L.K.); 3 Institute of Advanced Data Transfer Systems, ITMO University, Kronverkskiy Prospekt, 49, bldg. A, 197101 St. Petersburg, Russia; avkremleva@itmo.ru

**Keywords:** curcumin, electrospinning, ESKAPE, hyaluronic acid, pathogen, nanofibers, resistance

## Abstract

Bacterial infections have accompanied humanity throughout its history and became vitally important in the pandemic area. The most pathogenic bacteria are multidrug-resistant strains, which have become widespread due to their natural biological response to the use of antibiotics, including uncontrolled use. The current challenge is finding highly effective antibacterial agents of natural origin, which, however, have low solubility and consequently poor bioavailability. Curcumin, derived from *Curcuma longa*, is an example of a natural biologically active agent with a wide spectrum of biological effects, particularly against Gram-positive bacteria. However, curcumin exhibits extremely low antibacterial activity against Gram-negative bacteria. Curcumin’s hydrophobicity limits its use in medicine. As such, various polymeric systems have been used, especially biopolymer-based electrospun nanofibers. In the present study, the technological features of the fabrication of curcumin-loaded hyaluronic acid-based nanofibers are discussed in detail, their morphological characteristics, wettability, physico-chemical properties, and curcumin release profiles are demonstrated, and their antibacterial activity against multi-drug resistant ESKAPE pathogens (*Enterococcus faecium*, *Staphylococcus aureus*, *Klebsiella pneumoniae*, *Acinetobacter baumannii*, *Pseudomonas aeruginosa*, and *Enterobacter* species) are evaluated. It is noteworthy that the fibers containing a stable HA–curcumin complex showed high antibacterial activity against both Gram-positive and Gram-negative bacteria, which is an undeniable advantage. It is expected that the results of this work will contribute to the development of antibacterial drugs for topical and internal use with high efficacy and considerably lower side effects.

## 1. Introduction

Resistance to antimicrobial agents is a major global health threat. This threat is increasingly materialized through emerging resistance in many bacterial species. The problem is facilitated by antibiotic-induced selection in humans, animals, and agriculture; cross-species transfer of genetic mobile elements harboring resistance genes; and rapid global dispersion through transport and environmental waste. Meanwhile, although the R&D pipeline for new antibiotics is slowly refilling, only one in every four new antibiotics represents a novel drug class or mechanism of action. Moreover, none of these are active against the WHO-critical ESKAPE pathogens (*Enterococcus faecium*, *Staphylococcus aureus*, *Klebsiella pneumoniae*, *Acinetobacter baumannii*, *Pseudomonas aeruginosa*, and *Enterobacter* species) [1].

In February 2017, to focus and guide research and development related to new antibiotics, the World Health Organization (WHO) published its list of pathogens for which new antimicrobial development is urgently needed. Within this broad list, the so-called ESKAPE pathogens were designated priority status [2]. Many antibiotics in development are not active against the WHO-critical ESKAPE pathogens [2].

*Acinetobacter baumannii*, *Pseudomonas aeruginosa*, and *Enterobacteriaceae* belong to the first critical-priority category, and are resistant to carbapenems. The second priority category includes *Enterococcus faecalis* (resistant to vancomycin) and *Staphylococcus aureus* (resistant to methicillin and moderately sensitive to vancomycin). The WHO also published statistics for 2019 and identified 10 main causes of death (accounting for 55% of all registered deaths). Infectious diseases were three out of ten on this list and could be caused by bacterial strains of the ESKAPE group.

A systematic review of the clinical and economic impacts of antibiotic resistance revealed that ESKAPE pathogens are associated with the highest risk of mortality, thereby resulting in increased health care costs [3].

Moreover, in recent years, antibiotic resistance has increased due to the treatment of patients with a novel coronavirus infection COVID-19 [4].

The mechanisms of multidrug resistance exhibited by ESKAPE strains are broadly grouped into three categories, namely drug inactivation (commonly by an irreversible cleavage catalyzed by an enzyme), modification of the target site to which the antibiotic may bind, and reduced accumulation of drugs, either due to reduced permeability or by increased efflux of the drug [5]. They are also able to form biofilms that physically prevent the host’s immune response cells or antibiotics from inhibiting the pathogen. Moreover, biofilms protect specialized dormant cells, called persister cells, that are tolerant to antibiotics, leading to difficult-to-treat recalcitrant infections [6].

The overall number of antibiotics effective against ESKAPE strains is declining. This highlights an acute, urgent need for modern medications with novel mechanisms of action. The analysis of the antibiotic lists recommended in the Clinical & Laboratory Standards Institute (CLSI) guidelines revealed that many antibiotics suggested against ESKAPE strains since 2010 have been rejected, with the addition of relatively few new antibiotics or antibiotic combinations. Furthermore, there are incidences of resistance reported for some of these newly added antibiotics [7]. It is, therefore, imperative to find alternative ways to treat infections, especially those caused by ESKAPE pathogens.

In addition to the emergence of acquired bacterial resistance (against antibiotics currently in clinical use), another main concern is the overall variation of the human skin microbiota, which is related to the emergence of resistant microbial species induced by antibiotic agents [8]. This issue should limit topical and/or systemic long-term antibiotic therapies for the management of infectious diseases. In recent years, researchers have focused their attention on the development of plant-derived natural products as alternative or complementary options to traditional medicine. Indeed, the bioactive aromatic compounds obtained from some medicinal herbs have been shown to possess potential antimicrobial properties. In this context, the antimicrobial activity of curcumin has been extensively investigated due to its widespread use and good safety profile, even at high doses tested in clinical trials [9].

**Figure 1:**
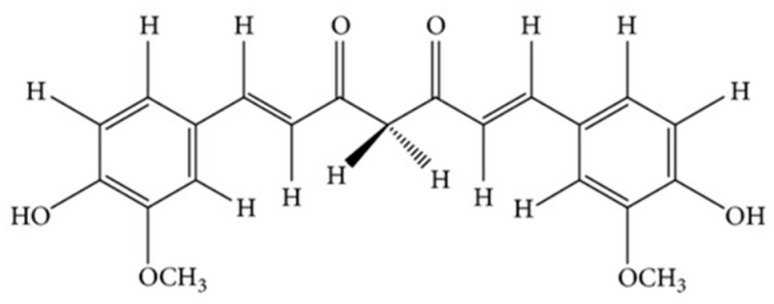
Chemical structure of curcumin.

Curcumin, also termed diferuloylmethane, has the preferred IUPAC name (1E,6E)-1,7-Bis (4-hydroxy-3-methoxyphenyl)-1,6-heptadiene-3,5-dione. Its structure is shown in Figure 1.

Since the identification of curcumin as the main component of the herbaceous plant turmeric, many studies have been carried out to evaluate the pharmacological properties of this substance. Based on the information from the PubMed database, since 2015, the number of articles devoted to the properties of curcumin has increased rapidly. The number of such publications has increased 16-fold when compared with an earlier time period (2000–2005). This shows the significant interest of the world’s scientific community in new substances with medicinal properties based on natural compounds.

The antibacterial properties of composites based on hyaluronic acid have been well documented [10,11]. Hyaluronic acid has been investigated regarding bacterial adhesion and biofilm formation; it has very moderate activity against biofilm formation and features notable anti-adhesive properties [12]. Dexamethasone (DE) is a well-known glucocorticoid drug. A recent publication presented the in vitro antibacterial activity of a composite hydrogel system (DE, HA, and chitosan) capable of inhibiting methicillin-resistant *Staphylococcus aureus* and *Escherichia coli* [13]. Compositions of curcumin with HA have been developed via covalent cross-linking [14,15,16]. A hyaluronic acid–curcumin covalently cross-linked conjugate shows antibacterial efficacy against *Staph. aureus* only when irradiated with light [17]. All of the above were considered in this study, which, for the first time, evaluated the antibacterial activity of curcumin-loaded nanofibers based on HA against multidrug-resistant ESKAPE group strains. We should emphasize that no cross-linking agents were used for the fabrication of curcumin-loaded nanofibers.

Studies have shown that, alone or in combination with other drugs, curcumin can enhance antibacterial, antifungal, and antitumor effects. Recently, the antimicrobial activity of curcumin has been proven against strains of *Staphylococcus aureus* (resistant to methicillin) and *Pseudomonas aeruginosa* (resistant to carbapenem), and the ability of curcumin to increase the effect of ciprofloxacin has been confirmed [18]. Studies have shown the potential use of curcumin as an additional component of medications for alleviating the symptoms of gastritis caused by the bacterium *Helicobacter pylori*. In addition, the analysis of antifungal activity showed the significant fungicidal effect of curcumin against *Candida* and *Paracoccidioides brasiliensis* [9].

It is known that HA is not active against *P. aeruginosa* [19,20,21]. There are controversial literature reports about the various effects of HA on *S. aureus*, such as growth promotion [22,23], no effect [19,20], or a clear inhibitory effect [24]. Only one publication demonstrated an effect of HA on *E. faecalis*. However, it was a delay in growth, rather than a prolonged inhibition [22]. The authors emphasized that the unexpected absence of activity against Gram-positive bacteria may be explained in various ways—either hyaluronidases are not produced, or the assessed HAs were not susceptible to microbial hydrolysis.

A formulation of covalently linked hyaluronan and poly-d,l-lactide, trademarked as ‘Defensive Antibacterial Coating’ (DAC^®^, Novagenit Srl, Mezzolombardo, Italy), was developed to protect various implants (orthopedics, traumatology, and dentistry) and maxillofacial surgical biomaterials from bacterial colonization [25,26]. It was shown that a vancomycin-loaded DAC^®^ coating was associated with a local bacterial load reduction ranging from 72 to 99%, compared with uncoated controls, in an acute model of highly contaminated implant-related infection in rabbit [27,28]. The effectiveness of DAC^®^ has been confirmed in clinical trials [29,30].

It has been reported that curcumin loading increases the antimicrobial effects of hyaluronic acid–PDMS (polydimethylsiloxane-diglycidyl ether) hydrogels. With *P. aeruginosa*, 39.28%, 57.14%, and 14% growth inhibition were seen in the presence of curcumin-loaded hydrogel A, curcumin-loaded hydrogel B, and HA–PDMS pure hydrogel, respectively [31].

The structure of curcumin enables the inhibition of bacterial growth by the formation of anti-oxidation products. It also suppresses the formation of bacterial biofilm and virulence factors, preventing adhesion to host receptors [32]. An in vitro test showed particular activity against certain species and strains: *Streptococcus pyogenes*, methicillin-resistant *Staphylococcus aureus*, *Acinetobacter lwoffii,* and certain strains of *Enterococcus faecalis* and *Pseudomonas aeruginosa*. However, the effectiveness of the substance is quite selective, depending on the microorganism type and strain [33].

However, curcumin’s solubility in water is limited (0.6 μg/mL), which is a serious problem due to its low bioavailability for the treatment of human diseases. In recent years, several approaches have been proposed to increase the bioavailability of curcumin without reducing its stability. The synergism of curcumin with antibiotics against *S. aureus* was demonstrated recently [34]. Curcumin nanostructures were developed based on cross-linked hyaluronic acid and zein polymers to improve their pharmacological properties. Such hybrids displayed improved features: increased stability, slowed decomposition of the drug, increased solubility, and improved pharmacokinetics. In studies of the antitumor activity of curcumin, the developed nanoparticles had increased therapeutic effects against triple-negative breast cancer cells MDA-MD-231. Even in combination with chemotherapeutic drugs, they elicited decreased expression of glycoprotein, a protein linked to breast cancer resistance [35].

This study evaluated the antibacterial activity of the developed curcumin-loaded nanofibers against polyresistant bacteria of the ESKAPE group: *Enterobacter cloacae*, *Staphylococcus aureus*, *Klebsiella pneumoniae*, *Acinetobacter baumannii*, *Pseudomonas aeruginosa*, and *Enterococcus faecalis*.

## 2. Materials and Methods

### 2.1. Materials

Hyaluronic acid (HA) sodium salt from *Streptococcus equi* (cosmetic grade, high molecular HA-T grade, MW 1290 kDa, HA 94.9%, glucuronic acid 45.9%, and protein 0.027%) was purchased from Bloomage Freda Biopharm Corporation Limited (Jinan, China). Dimethyl sulfoxide (DMSO, 99.5% ACS, MW = 78.13 g/mol) was obtained from JSC EKOS-1 (Moscow, Russia). Curcumin powder from *Curcuma longa* (Turmeric) (MW = 368.38 g/mol, assay percentage 95.0%), phosphate-buffered saline (PBS, pH 7.4), and ethanol were obtained from Sigma-Aldrich (St. Louis, MO, USA). All chemicals were used without additional purification. Distilled water was obtained using distilling apparatus.

Hyaluronic acid (HA) sodium salt was chosen as a polymer basis for nonwoven nanofibrous materials obtained by the electrospinning technique. DMSO was used as a co-solvent. Curcumin of natural origin was used as the biologically active agent due to its reported unique set of antibacterial, antiviral, and anti-inflammatory properties. Spunlace (viscose:polyether 1:1) was obtained from LLC “Elegreen” (St. Petersburg, Russia) and used as the subsurface for nanofibers.

### 2.2. Microbial Strains and Culture Media

The antimicrobial activity of curcumin nanofibers was investigated against the growth of ESKAPE pathogens: Enterobacter cloacae, Staphylococcus aureus, Klebsiella pneumoniae, Acinetobacter baumannii, Pseudomonas aeruginosa, and Enterococcus faecalis (*n* = 8). We used the following reference strains: Enterobacter cloacae NCTC 13406, Staphylococcus aureus ATCC 29213, Klebsiella pneumoniae ATCC 13883, Acinetobacter baumannii ATCC 19606, Pseudomonas aeruginosa ATCC 27853, and Enterococcus faecalis ATCC 29212. The sources of the bacterial strains are listed in Table 1. The experiments to evaluate the antibacterial properties were performed in accordance with the recommendations of the Clinical and Laboratory Standards Institute (CLSI) [36] and according to the European Committee on Antimicrobial Susceptibility Testing (EUCAST) [37]. Mueller Hinton Agar (MHA) and Columbia agar + 5% sheep blood were sterilized by autoclaving (121 ± 2 °C, 103 ± 5 kPa) for 30 min. Warm medium (20 mL) was poured into Petri dishes (Nunc, Denmark) and allowed to cool at ambient temperature. The inocula for MIC were prepared in sterile Mueller–Hinton broth (Oxoid). The microorganisms were grown at 37 °C for 24 h. Analyses were performed using five experiments for each of the analyzed samples.

### 2.3. Formation of Polymer Solutions

Hyaluronic acid sodium salt HA-T was dissolved in a distilled water/DMSO binary (equivoluminar) solvent mixture to obtain a solution with a polymer concentration equal to 19.0 mg/mL [38]. The forming polymer solutions were mixed under ambient conditions by magnetic stirring. Curcumin was added directly to the spinning polymer solutions with the weight ratios of biopolymer to curcumin as follows: 1:0, 25:1, 15:1, and 5:1. Note that additional chemical agents, such as 4-dimethylaminopyridine (DMAP) or 1,3-dicyclohexylcarbodiimide (DCC), were not used for the solutions’ preparation.

### 2.4. Electrospinning

**Figure 2:**
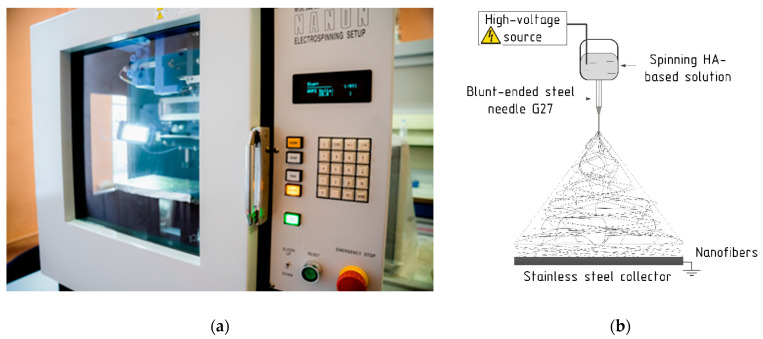
(**a**) Universal electrospinning setup NANON-01A; (**b**) Illustrative scheme of the electrospinning process.

Nanofibrous membranes were obtained by the electrospinning technique using universal equipment NANON-01A (MECC CO., LTD., Fukuoka, Japan, Figure 2a).

The method of nanofiber production by electrospinning and the principal scheme of this process were detailed in the previous publication [39]. Briefly, each polymer solution was individually loaded into a 5 mL syringe (Luer-Lock type), which was connected by a Teflon tube and a special metal connector to a blunt-ended steel needle, through which the electrohydrodynamic jetting of the polymer solution was carried out. An illustrative scheme of the electrospinning process is shown in Figure 2b.

The technological parameters of the electrospinning process are listed in Table 2.

### 2.5. Morphology and Diameters of Nanofibers

A MIRA3 TESCAN scanning electronic microscope (SEM) was used for precision determination of the morphology and size distribution of electrospun curcumin-loaded nanofibers based on hyaluronic acid. The parameters were: acceleration voltage 10 kV, SE detector, and working distance 7.84–8.07 mm. The samples were scanned after coating with 3 nm gold using a Quorum Q150R S/E/ES Plus coating system.

### 2.6. Digital and Statistical Analysis

For the digital analysis of photomicrographs and diameter measurement, the program ImageJ (National Institutes of Health, Bethesda, MD, USA) was used [40]. For each nanofiber sample, 400 measurements were taken using 2–3 SEM photomicrographs.

The diameter distribution was determined using OriginPro software (OriginLab Corporation, Northampton, MA, USA). Several photographs were used for statistical analysis.

### 2.7. Wettability

The water contact angles of the HA-based nanofibers were measured with a Drop Shape Analyzer KRÜSS DSA 100R (KRÜSS GmbH, Hamburg, Germany) using the sessile drop technique (2 μL drop of distilled water). Each sample was analyzed five times on various parts of the nanofibers’ surfaces. The water contact angles were measured at the 0, 10th, 20th, 30th, and 60th seconds.

### 2.8. Fourier-Transform Infrared Spectroscopy (FTIR)

Fourier-transform infrared spectra (FTIR) were recorded using a Tensor 37 spectrometer (Bruker Optik GmbH, Germany). The parameters were as follows: ambient temperature 21 ± 1.0 °C, frequency range 4000–600 cm^−1^, and spectral resolution 2 cm^−1^.

### 2.9. Thermogravimetric Analysis (TGA) 

The study of the properties of the initial components, as well as the obtained nanofibers, was carried out using thermogravimetric analysis (TGA). The measurements were obtained using a TG 209 F1 Libra vacuum-tight micro-thermal balance (NETZSCH, Germany).

The parameters were as follows: temperature range from 25 °C to 900 °C, heating rate 10 °C/min, nitrogen atmosphere (gas flow rate 40 mL/min), and Al_2_O_3_ measuring pan material.

### 2.10. Differencial Scanning Calorimetry (DSC)

DSC analysis was carried out by using a NETZSCH DSC 204 F1 Phoenix Differential Scanning Calorimeter (NETZSCH, Germany). The measurement parameters were as follows: temperature range from −30 to 210 °C, heating rate 10 °C/min, aluminum pans, flow of nitrogen gas equal to 40 mL/min, and empty pan as the reference cell. DSC analysis was conducted for blank HA-based nanofibers, curcumin-loaded HA-based nanofibers, mechanical mixtures of HA and curcumin powders with the weight ratios of biopolymer to curcumin equal to 25:1 and 5:1, and for pure curcumin powder.

### 2.11. Drug Loading and Encapsulation Efficiency 

Accurately weighed nanofiber samples were dissolved in 10.0 mL equivoluminar solution of phosphate-buffered saline (PBS, pH 7.4) and ethanol. After dissolution, the solution was analyzed using a UV spectrophotometer UNICO UV-2804 (USA) at 429 nm from the previously obtained calibration curve for curcumin in the same media. The drug-loading capacity (*DL*) and encapsulation efficiency (*EE*) were calculated using the formulae [41,42]:

(1) $$DL=\frac{Amount\;of\;curcumin\;in\;the\;HA-based\;nanofibers}{Amount\;of\;the\;HA-based\;nanofibers}\times 100$$

(2) $$EE=\frac{Amount\;of\;curcumin\;in\;the\;HA-based\;nanofibers}{Amount\;of\;the\;initial\;curcumin}\times 100$$

For each curcumin-loaded nanofiber, the determinations were carried out in triplicate and the results are presented with the standard deviation (SD).

### 2.12. In Vitro Drug-Release Study

The kinetics of curcumin release from polymer nanofibers were determined in the release medium consisting of PBS (pH 7.4) and ethanol with a volume ratio of 1:1 [43] and volume equal to 10.0 mL. The accurately weighed electrospun curcumin-loaded HA-based nanofibers were immersed in the release solution with constant shaking at 100 rpm and 37 ± 1 °C. An aliquot (2.0 mL) from each sample was taken for UV analysis at the determined time intervals and 2.0 mL of the fresh release medium was added to maintain the same total volume. The actual curcumin concentration in the release medium was detected as in actual drug content analysis (Section 2.11.). The release kinetics of curcumin from the nanofibers were analyzed over a period of 120 min, after which the nanofibers had totally dissolved. Each type of curcumin-loaded nanofiber was analyzed in triplicate.

### 2.13. Antimicrobial Activity

The microbial growth inhibition potential of the nanofibers was determined by using the agar-disk-diffusion method according to the Clinical and Laboratory Standards Institute (CLSI) recommendations [36].

Each freshly grown bacterial strain (24 h growth) from the plates was suspended in sterile saline at a density of 1.0 OD600 (approximately 5 × 10^8^ CFU/mL). For susceptibility testing by the disk-diffusion method, the commercial MHA and Columbia agar plates were inoculated with 100 μL of the suspension (approximately 5 × 10^7^ CFU). Using a sterile cotton swab, the inoculum was spread evenly over the entire surface of the CBA plate by swabbing in three directions.

For the disk-diffusion method, nanofibers were cut into 10 × 10 mm^2^ shreds and sterilized for 30 min (125 °C, 103 KPa). They were then placed on the surface of the inoculated plates (6 nanofibers per plate). After incubation at 37 °C for 1 day, the inhibition zone diameters were measured in millimeters (mm) in accordance with the reading guide of the EUCAST disk-diffusion method. The plates were read (30 cm from the eye) from the front with the lid removed using reflected light illumination. In case of distinct colonies within the zone, the diameter was measured within the closest colony to the disk. The inhibition zone diameter was recorded as 6 mm when the bacteria grew in contact with the nanofiber’s edge. The experiments were repeated three times.

The minimum inhibitory concentrations (MICs) were determined by the broth microdilution method using 96-well plates (Nunc). Briefly, 50 µL of Mueller–Hinton broth (Oxoid) was placed in each well. The stock solution of nanofibers was placed in the first well (50 µL), and serial dilutions were performed. For the disk-diffusion method, we dissolved 3 pieces of 10 × 10 mm nanofibers containing curcumin and HA in 2 mL of sterile saline (exposition 24 h); after that, we conducted an experiment with a stock solution at a concentration of 720 mg/mL. The subsequent dilutions for the determination of MICs were carried out twice. Further, we bred the bacteria in a nutrient medium with a content of 360 mg/mL. The inocula were adjusted to contain approximately 10^8^ CFU/mL bacteria. Bacterial inocula (50 µL) were added to the wells. Next, the plates were incubated at 37 °C for 24 h. The MIC value was calculated as the lowest concentration of the extract that inhibited any visible bacterial growth. In addition, the seeding was counted from each well onto dense nutrient medium, and the number of CFUs was counted after incubation. A second growth measurement was assessed by the change in color of the nutrient medium with the colorant resazurin. A third measurement was obtained by analyzing the solution density using an optical microplate reader (Elisa Plate Reader).

## 3. Results and Discussion

A number of authors achieved a bactericidal effect against *E. faecalis* using curcumin only through the use of LED photodynamic therapy [44]. In our study, this was not required due to the use of hyaluronic acid-based nanofiber technology loaded with curcumin. The method of applying the thinnest film with an antibacterial effect to a wound’s surface is more convenient when used on large surfaces, including for maintaining sterility in operating units. The concentration of 90 μg/mL was quite suitable for the use of the drug on large areas.

Some authors have achieved an antibacterial effect against P. aeruginosa bacteria using curcumin nanoparticles. However, the effect was expressed in relation to planktonic forms of microorganisms [45]. However, it is known that the greatest danger is not so much planktonic as biofilm forms of these bacteria. Therefore, the use of nanofiber coatings with curcumin will be in demand for the treatment of wound surfaces, especially for burn units.

An interesting development based on chitosan nanofibers with curcumin has also shown good results against S. aureus strains [46]. However, we must not forget that, in practice, allergic reactions to chitosan are often observed, so the range of possible use of hyaluronic acid is much wider.

The most dangerous microorganism currently leading to the development of severe bacterial complications in pneumonia of various origins is K. pneumoniae. The authors of publication [47] showed the possibility of eliminating this pathogen directly from the lungs in experiments on animals in vivo. To do so, they used concentrations of curcumin comparable to those found to be effective in our study.

### 3.1. Nanofibers Characterization

**Figure 3:**
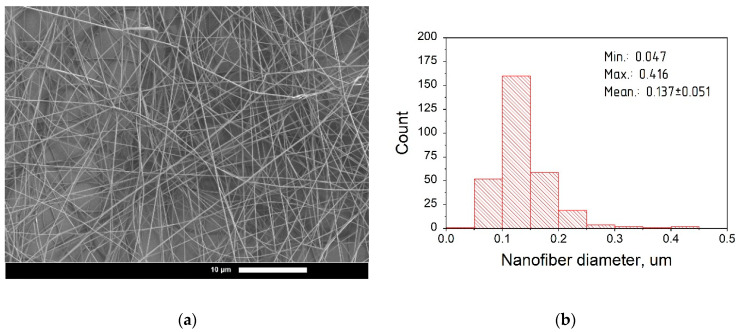
(**a**) SEM photomicrographs of blank hyaluronic acid electrospun fibers (magnification 4000×); (**b**) Diameter distribution of fibers based on hyaluronic acid.

**Figure 4:**
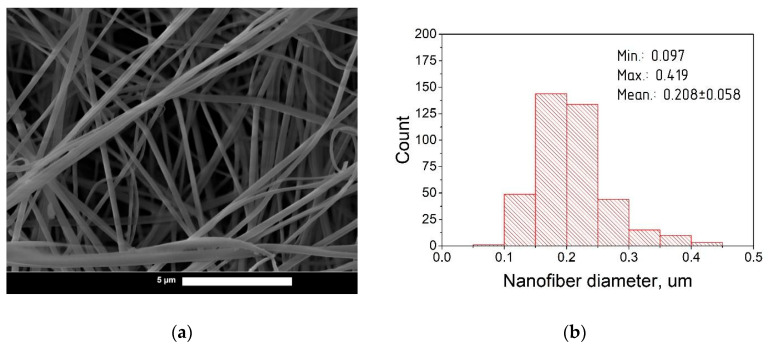
(**a**) SEM photomicrographs of curcumin-loaded hyaluronic acid electrospun fibers (HA:curcumin 25:1; magnification 47,600×); (**b**) Diameter distribution of curcumin-loaded fibers based on hyaluronic acid.

**Figure 5:**
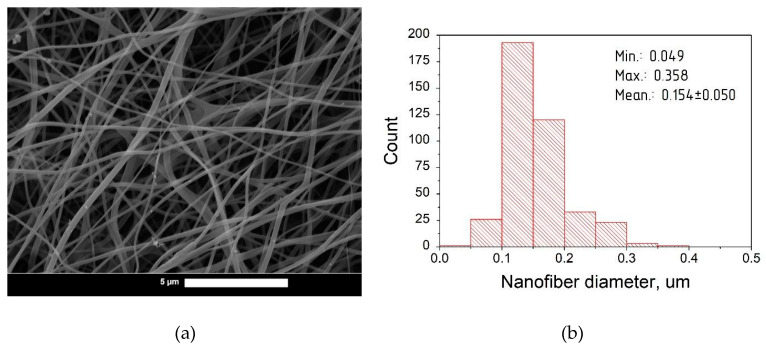
(**a**) SEM photomicrographs of curcumin-loaded hyaluronic acid electrospun fibers (HA:curcumin 15:1; magnification 44,400×); (**b**) Diameter distribution of curcumin-loaded fibers based on hyaluronic acid.

**Figure 6:**
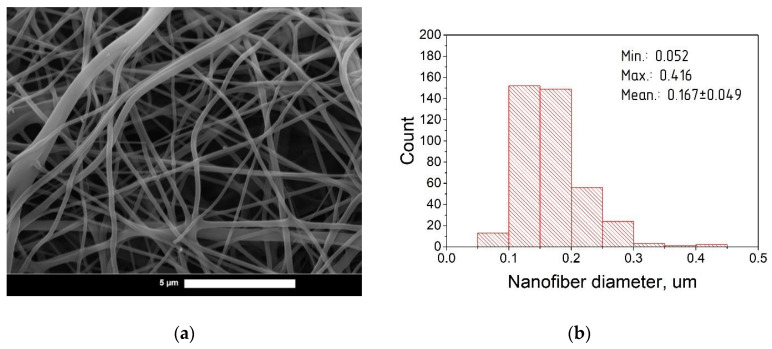
(**a**) SEM photomicrographs of curcumin-loaded hyaluronic acid electrospun fibers (HA:curcumin 5:1; magnification 47,600×); (**b**) Diameter distribution of curcumin-loaded fibers based on hyaluronic acid.

Nanofibers based on hyaluronic acid with a range of curcumin contents were characterized by scanning electron microscopy (SEM) and statistical analysis. Photomicrographs and diameter distribution histograms of curcumin-loaded nanofibers with HA:curcumin weight ratios of 1:0, 25:1, 15:1, and 5:1 are depicted in Figure 3, Figure 4, Figure 5 and Figure 6, respectively.

The summarized information about the morphological characteristics of native and curcumin-loaded hyaluronic acid nanofibers is shown in Table 3. Native nanofibers based on hyaluronic acid were analyzed in detail in our previous publication [48]. Diameter distributions were evaluated using ImageJ software based on several SEM images.

As shown in Table 3, the average diameter initially increased after the addition of curcumin to the HA solution. This effect may be associated with the filling nature of curcumin as an additional component in the spinning solution. The increase in the average diameter after curcumin incorporation has also been demonstrated in previous studies [49,50,51].

Interestingly, with the increasing curcumin content, the average fiber diameter decreased. It is known that addition curcumin to the spinning solution also increases the electrical conductivity [51,52]. This leads to the better surface charge transfer from the electrode to the polymer solution, resulting in the formation of nanofibers with a smaller average diameter due to the more efficient splitting of the jet [53]. A similar effect was detected in previous studies [53,54]. At the same time, with the 5:1 ratio (HA:curcumin, by weight), a slight increase in the average diameter was detected; this can be attributed to the greater filling effect with curcumin over its stabilizing action on electrospinning [38].

### 3.2. Wettability

**Figure 7:**
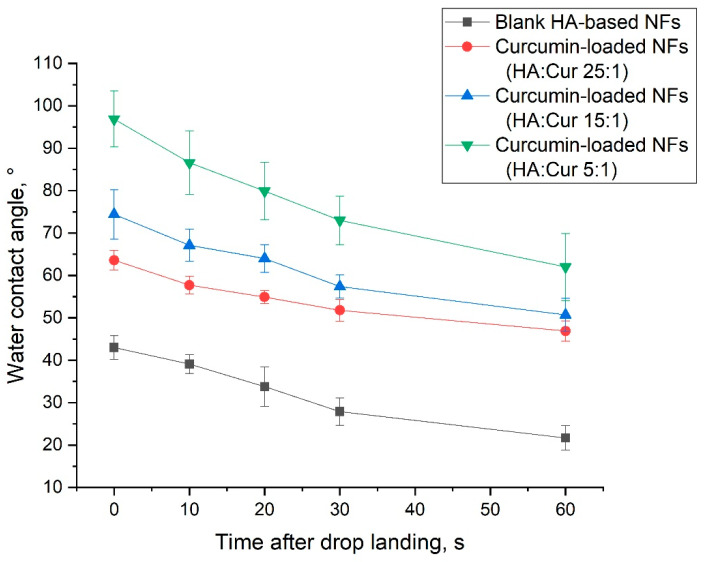
Water contact angles vs. time of distilled water on electrospun nanofibers based on hyaluronic acid loaded with various contents of curcumin: HA:curcumin ratio (by weight) 1:0; HA:curcumin ratio (by weight) 25:1; HA:curcumin ratio (by weight) 15:1; HA:curcumin ratio (by weight) 5:1.

To analyze the effect of curcumin loading and its content on the wettability of the HA-based electrospun nanofibers, the water contact angles were measured (Table 4 and Figure 7).

As shown in Table 4 and Figure 7, the native HA-based nanofibers demonstrated a hydrophilic surface with a contact angle equal to 43.0° ± 2.8° at the beginning. The addition of curcumin enhanced the water contact angle and changed the hydrophilicity of the HA-based nanofibers to hydrophobicity: the water contact angle increased to 63.6° ± 2.3°, 74.4° ± 5.8°, and 96.9° ± 6.6°. Thus, curcumin-loaded nanofibers with the maximum curcumin content had a hydrophobic surface, which was significantly higher than that of nanofibers based on native hyaluronic acid. In general, the tendency observed corresponds to that in similar studies [55,56].

### 3.3. Fourier-Transform Infrared Spectroscopy (FTIR)

**Figure 8:**
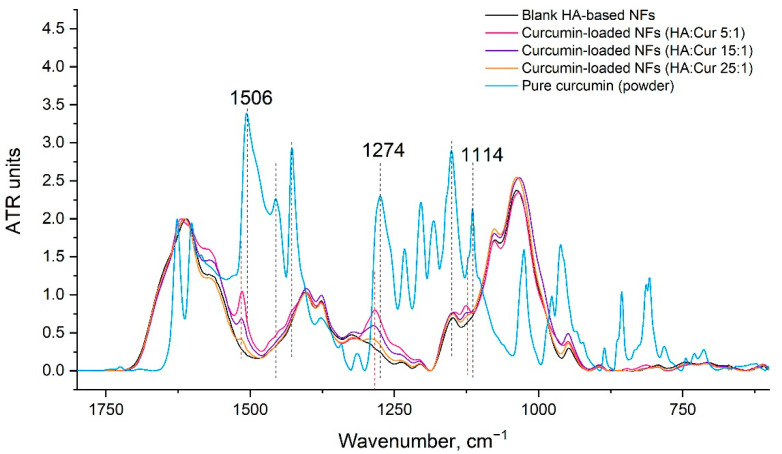
FTIR spectra of blank HA-based nanofibers (NFs), curcumin-loaded HA-based nanofibers with different HA:curcumin ratios: 25:1, 15:1, and 5:1 (by weight); mechanical mixtures of pure HA and curcumin with HA:curcumin ratios equal to 25:1 and 5:1 (by weight), and pure curcumin powder.

The formation of the curcumin–HA complex could be confirmed by the FTIR spectra of the obtained nanofibers based on HA with different contents of the biologically active substance (Figure 8). Thus, the FTIR spectrum of curcumin has stretching vibrations due to phenolic hydroxyl groups at 3200–3500 cm^−1^, stretching vibrations at 1628 cm^−1^ associated mainly with overlapping stretching vibrations of C=C double bonds and carbonyl groups C=O, aromatic stretching vibration C=C at 1427 cm^−1^, and a high-intensity band at 1504 cm^−1^ related to mixed vibrations, including carbonyl bond stretching ν (C=O), in planar bending vibrations around aliphatic δC-C-C, δC-C=O, and in planar bending vibrations around the aromatic δCC-H keto and enol configurations, and stretching vibrations around the aromatic νCC bonds of the keto- and enol- forms of curcumin. In addition, a significant intense band at 1274 cm^−1^ was attributed to the bending vibration of the phenol band ν (C-O), and that at 1114 cm^−1^ to the vibrations of the C-O bonds.

In the HA-based nanofibers, an increase in the absorption bands belonging to curcumin was observed with an increase in its proportion in the nanofibers. However, the curcumin bands were shifted in the fibers in comparison with the position of the curcumin powder bands, indicating the formation of a complex with HA (1540 was shifted by 1514 cm^−1^, 1427 was shifted by 1283 cm^−1^, and 1114 was shifted by 1126 cm^−1^).

### 3.4. Thermogravimetric Analysis (TGA)

**Figure 9:**
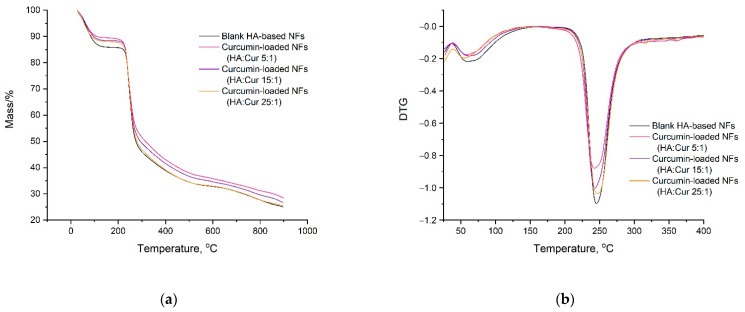
TGA thermograms (**a**) and derivative thermogravimetric (DTG) curves (**b**) of blank HA-based nanofibers (NFs) and curcumin-loaded HA-based nanofibers with various HA:curcumin ratios of 5:1, 15:1, and 25:1 (by weight).

As seen from the data obtained by the TGA of curcumin-containing hyaluronic acid-based fibers (Figure 9), the low mass loss (2–3%) of the tested samples in the region of boiling point of DMSO 189 °C indicated the almost complete absence of organic solvent in the fibers, which is an important fact for practical applications in medicine. The weight loss (10–14%) corresponded to the evaporation of water molecules that bonded to hyaluronic acid.

The temperature values corresponding to the weight losses of 10 and 50% polymer fibers obtained from solutions with a HA concentration of 1.9 wt.% in an equal-volume mixture of distilled water and DMSO with different curcumin contents are presented in Table 5.

With the increase in the curcumin content of the polymer matrix, the temperature resistance of the nanofibers increased, which may have indicated the formation of a strong and stable complex of curcumin with hyaluronic acid. The thermal stability of the curcumin-loaded HA-based nanofibers increased by an average of 10–30 °C with an increase in the curcumin content by 1.6–3.0 times.

It should be noted that increase in the curcumin content in the nanofibers slightly decreased the moisture content of the materials determined by TGA: HA_0 = 14.2%, HA_25:1 = 12.2%, HA_15:1 = 11.6%, and HA_5:1 = 10.6%. This was probably caused by the decrease in the number of water molecules involved in the hydrogen-bonding stabilization of the three-dimensional structure of HA due to the formation of the HA–curcumin complex.

### 3.5. Differencial Scanning Calorimetry (DSC)

**Figure 10:**
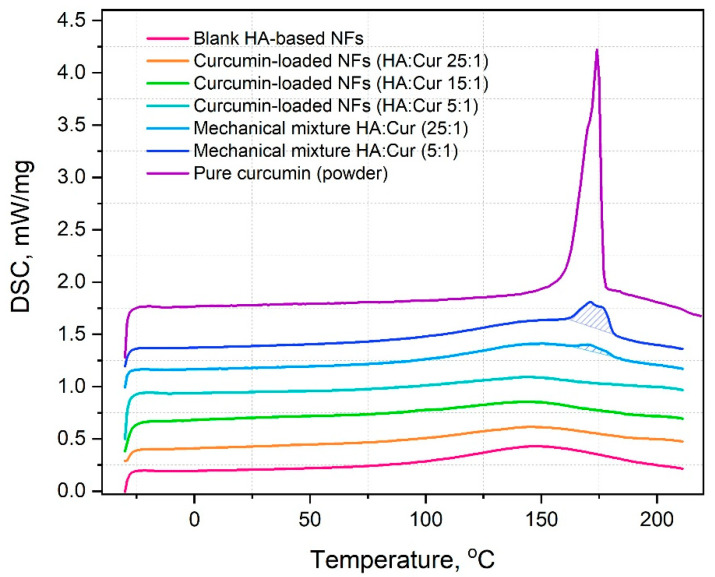
DSC thermograms of (from the bottom upwards): blank HA-based nanofibers (NFs), curcumin-loaded HA-based nanofibers with different HA:curcumin ratios of 25:1, 15:1, and 5:1 (by weight); mechanical mixtures of pure HA and curcumin with HA:curcumin ratios of 25:1 and 5:1 (by weight), and pure curcumin powder.

Figure 10 demonstrates the thermal properties of the electrospun HA-based nanofibers, mechanical mixtures of HA and curcumin powders, and the pure curcumin powder. The key difference between the mechanical mixtures of HA/curcumin and curcumin-loaded nanofibers was observed at 175 °C, which was attributed to the melting peak of curcumin. HA nanofibers and curcumin-loaded nanofibers had no characteristic phase transitions in the temperature range of −30–210 °C. In comparison, the thermal profiles of the mechanical mixtures of curcumin and HA had an endothermic peak corresponding to the curcumin melting point. Such an effect confirms the successful loading of curcumin into the polymeric matrix and the formation of a stable complex.

### 3.6. Drug Loading and Encapsulation Efficiency 

The results of the drug-loading capacity (*DL*) and encapsulation efficiency (*EE*) analyses are presented in Table 6.

According to the Table 6, the encapsulation efficiency was similar for all curcumin-loaded HA-based nanofibers. However, the values differed from 100% due to the formation of curcumin–HA complexes, which could not be formed without the residual amounts of DMSO and H_2_O.

The drug-loading capacity directly depended on the initial curcumin amount and increased with the curcumin content increase. At the same time, a further increase in the drug content of the polymer system caused the participation of curcumin; thus, a mass ratio of drug and HA equal to 1:5 could be considered as ultimate. The difference between the theoretical values of *DL* could also be explained by the presence of water and DMSO residuals, which were included in the curcumin–HA complex.

### 3.7. In Vitro Drug-Release Study

**Figure 11:**
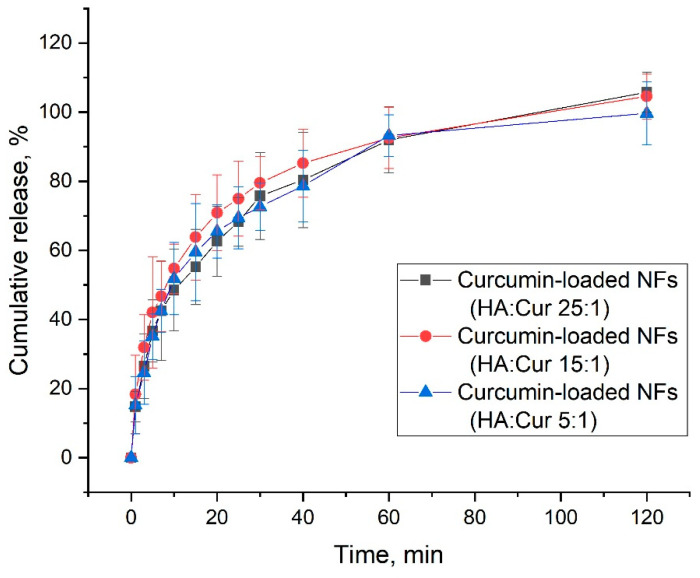
Curcumin cumulative release profiles from curcumin-loaded HA-based nanofibers (NFs).

The curcumin release profiles into PBS:ethanol media (volume ratio equal to 1:1) from HA-based electrospun nanofibers are shown in Figure 11. The profiles had similar kinetics, regardless of the curcumin content in the nanofibers. Note that there were no burst effects—curcumin gradually dissolved in the release media over 120 min. Such release profiles allow the use of the nanofiber obtained as a potential antibacterial wound dressing with gradual drug release and rapid relief of inflammation and bacteria.

### 3.8. Antimicrobial Activity of Curcumin-Loaded Nanofibers Based on Hyaluronic Acid

**Figure 12:**
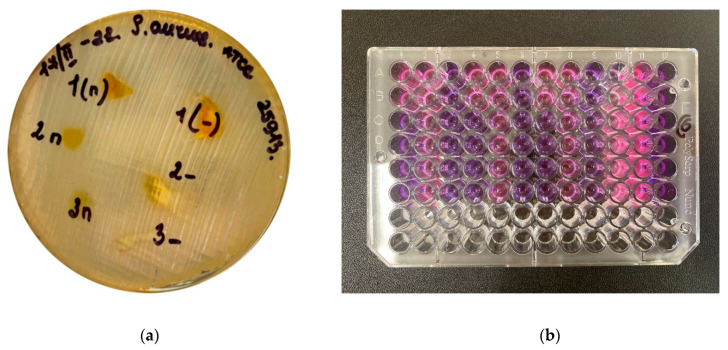
(**a**) Culture of *Staphylococcus aureus* strain with a visible zone of growth inhibition (ZGI) around the nanofibers; (**b**) Representative image of antibacterial activity by a colorimetric assay (MIC) for *Pseudomonas aeruginosa*.

In this research, the activity of the curcumin-loaded nanofibers against bacterial strains belonging to six species was evaluated. A clearly stronger effect against Gram-positive than Gram-negative bacteria was observed. The median zones of growth inhibition (ZGIs) for Gram-positive bacteria were between 9.0 and 19.0 mm, while those for Gram-negative bacteria ranged from 6.0 to 14.0 mm. The minimal inhibitory concentrations (MICs) for Gram-positive bacteria reached values of about 90 µg/mL (median 90 µg/mL), while those for Gram-negative bacteria ranged from 90 to 360 µg/mL (median 360 µg/mL) (Figure 12).

**Figure 13:**
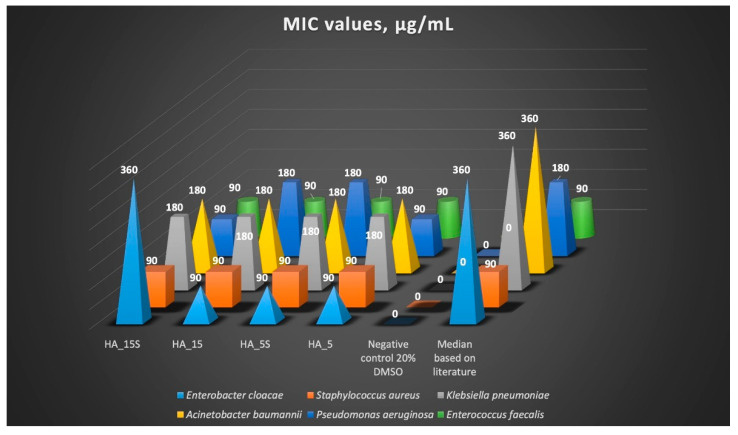
Schematic representation of the mean minimum inhibitory concentration (MIC) values (µg/mL).

The minimal inhibitory concentrations (MICs) were determined for the leading (best scoring) nanofibers (HA_15 s, HA_15, HA_5 s, and HA_5). The data presented in Table 7 demonstrate that curcumin-loaded nanofibers exhibited the greatest antibacterial activity against Gram-positive bacteria. The suppression of Gram-negative bacteria under the action of curcumin and curcumin-loaded fibers is demonstrated in this work for the first time. The obtained values for the zones of growth inhibition and MICs are presented in Table 7 and Table 8 and Figure 13.

It has been found that the MIC of curcumin against *Staphylococcus aureus* ATCC 29213 fluctuates at the level of 219 µg/mL [57]; this is notably higher than that obtained for curcumin-loaded fibers based on HA studied in this work—90 µg/mL.

The MICs of curcumin against strains of *E. faecalis* ATCC 51299 and *E. faecalis* ATCC 29212 were determined to be between 156 µg/mL and 625 µg/mL [58,59], and in our experiment, it was notably lower—90 µg/mL.

In several studies, the MIC values of curcumin against *P. aeruginosa* ATCC 27853 were determined to range from 30–512 µg/mL [60]. In this study, the MIC of curcumin-loaded fibers against *P. aeruginosa* ATCC 27853 was determined to be about 90–180 µg/mL. Large MIC values for curcumin were also noted against our clinical *K. pneumoniae* and *A. baumannii* strains: more than 2000 µg/mL and 5000 µg/mL, respectively [33]. With the reference *K. pneumoniae* and *A. baumannii* strains, we found significantly lower MIC values (360 µg/mL).

## 4. Conclusions

The multi-drug resistance of bacterial infections is a global problem around the world that requires novel drugs with new mechanisms of action. This problem is urgent, especially in the era of pandemics. In recent years, the antibacterial activity of curcumin against Gram-positive strains has been demonstrated in open potential application in the fields of infection prevention and control. Specially developed drug delivery systems incorporating antimicrobials further expand these possibilities and overcome restrictions for the medical use of water-insoluble compounds of natural origin. This study evaluated the antibacterial properties of polymer nanofibers based on hyaluronic acid with curcumin fabricated by the electrospinning process. Gram-negative bacteria have been predominant among nosocomial infections recently. In this paper, we demonstrated for the first time the formation of a stable complex between curcumin and hyaluronic acid without cross-linking agents. The wettability, physico-chemical properties, and curcumin-release kinetics were also demonstrated for the first time, as well as the ability of curcumin-loaded nanofibers based on hyaluronic acid to be active against both Gram-positive and Gram-negative strains. The inhibitory activity of the developed fibers carries significant medical potential as candidate antibacterial materials in several scenarios where the control of microbial colonization/expansion is required (prophylaxis, external infections, superficial trauma, deep tissue trauma, etc.). These features match the well-known, real need for the creation of highly effective antibiotics (or related materials) based on natural biologically active substances with minimal side effects.

## Figures and Tables

**Table 1:** Sources of bacterial strains (*n* = 8).

Organism.	Source
*Enterobacter cloacae*	NCTC 13406
*Staphylococcus aureus*	ATCC 29213
*Klebsiella pneumoniae*	ATCC 13883 and clinical strain
*Acinetobacter baumannii*	ATCC 19606 and clinical strain
*Pseudomonas aeruginosa*	ATCC 27853
*Enterococcus faecalis*	ATCC 29212

**Table 2:** Technological parameters of electrospinning.

Technological Parameter	Value
Applied voltage	28.0 kV
Volume flow rate	2.0 mL/h
Spinneret speed	10.0 mm/s
Steel needle size (Gauge scale)	27 G
Syringe volume	5.0 mL
Collecting electrode (width × length)	150 × 200 mm
Distance between the needle and electrode	150 mm
Subsurface for nanofiber membranes	Silicon plates 10 × 10 × 1 mm (for microscopy) Spunlace, 150 × 200 mm (for antibacterial assay)
Process time	5 min (samples for microscopy) 40 min (samples for antibacterial assay)
Drying time	10 min
Temperature	23.0 ± 2.0 °C
Relative humidity	20.0–25.0%

**Table 3:** Morphological characteristics of the nanofibers obtained.

Sample Name	HA: Curcumin Ratio (by Weight)	Diameter of Fibers Obtained (nm)		
		Min	Max	Mean
HA_0	1:0	47	416	137 ± 51
HA_25	25:1	97	419	208 ± 58
HA_15	15:1	49	358	154 ± 49
HA_5	5:1	52	416	167 ± 50

**Table 4:** Measured water contact angles of HA-based nanofibers.

Scheme	HA: Curcumin Ratio (by Weight)	Water Contact Angle After Various Periods of Drop Landing				
		0 s	10 s	20 s	30 s	60 s
HA_0	1:0	43.0 ± 2.8	39.1 ± 2.2	33.8 ± 4.7	27.9 ± 3.2	21.7 ± 2.9
HA_25	25:1	63.6 ± 2.3	57.7 ± 2.1	54.9 ± 1.5	51.8 ± 2.6	46.9 ± 2.4
HA_15	15:1	74.4 ± 5.8	67.1 ± 3.8	64.0 ± 3.3	57.4 ± 2.7	50.7 ± 3.9
HA_5	5:1	96.9 ± 6.6	86.6 ± 7.5	79.9 ± 6.8	73.0 ± 5.7	62.0 ± 7.9

**Table 5:** Temperature values corresponding to weight losses of 10 and 50%.

Sample Name	HA: Curcumin Ratio (by Mass)	Weight Loss, %	T, °C
HA_0	1:0	10	89
		50	277
HA_25	25:1	10	88
		50	279
HA_15	15:1	10	96
		50	294
HA_5	5:1	10	110
		50	315

**Table 6:** *DL* and *EE* of HA-based nanofibers (*n* = 3, ± SD).

Sample Name	HA:curcumin Ratio (by Mass)	Drug-Loading Capacity (*DL*), %		Encapsulation Efficiency (*EE*), %	
		Theoretical	Experimental	Theoretical	Experimental
HA_25	25:1	3.8	2.8 ± 0.6	~100.0	70.0 ± 1.8
HA_15	15:1	6.3	4.6 ± 0.7	~100.0	69.5 ± 2.1
HA_5	5:1	16.7	14.6 ± 1.1	~100.0	72.8 ± 1.9

**Table 7:** Microbial growth inhibition zones (mm).

Simple Name	ESKAPE Group Strains					
	*E. Cloacae* (−)	*S. Aureus* (+)	*K. Pneumoniae* (−)	*A. Baumannii* (−)	*P. Aeruginosa* (−)	*E. Faecalis* (+)
HA_0 s	6	6	6	6	6	6
HA_0	6	6	6	6	6	6
HA_25 s	6	9	6	6	6	6
HA_25	6	19	6	6	9	6
HA_15 s	6	13	6	6	11	9
HA_15	13	10	6	6	13	10
HA_5 s	9	15	6	6	14	13
HA_5	7	11	6	6	11	10
Negative control (20% DMSO)	6	6	6	6	6	6

Note: index “s” in the sample name means the subsurface “Spunlace”.

**Table 8:** Mean minimum inhibitory concentration (MIC) values (µg/mL).

Sample Name	ESKAPE Group Strains					
	*E. Cloacae*	*S. Aureus*	*K. Pneumoniae*	*A. Baumannii*	*P. Aeruginosa*	*E. Faecalis*
HA_15 s	360 (3×)	90 (2×), 180	180, 360 (2×)	180, 360 (2×)	90, 180 (2×)	90 (3×)
HA_15	90 (3×)	90 (3×)	180, 360 (2×)	180, 360 (2×)	180, 360 (2×)	90 (3×)
HA_5 s	90 (3×)	90 (3×)	180, 360 (2×)	180, 360 (2×)	180, 360 (2×)	90 (3×)
HA_5	90 (3×)	90 (2×), 180	180, 360 (2×)	180, 360 (2×)	90, 180 (2×)	90 (3×)
Negative control (20% DMSO)	-	-	-	-	-	-
Median	360	90	360	360	180	90

Note: index “s” in the sample name means the subsurface “Spunlace”.

## Data Availability

Not applicable.
